# From Farm to Finger Prick—A Perspective on How Plants Can Help in the Fight Against COVID-19

**DOI:** 10.3389/fbioe.2020.00782

**Published:** 2020-07-03

**Authors:** Karen A. McDonald, R. Barry Holtz

**Affiliations:** ^1^Department of Chemical Engineering, University of California, Davis, Davis, CA, United States; ^2^Food and Pharmaceutical Synthesis Division Lead, Center for the Utilization of Biological Engineering in Space, Berkeley, CA, United States; ^3^Global HealthShare® Initiative, Davis, CA, United States; ^4^Holtz Biopharma Consulting, Austin, TX, United States; ^5^Scientific Advisory Committee, Center for the Utilization of Biological Engineering in Space, Berkeley, CA, United States

**Keywords:** COVID-19, plant made pharmaceuticals, diagnostic test, SARS-CoV-2, crops, plant made SARS-CoV-2 antigens

## Abstract

As a consequence of the COVID-19 pandemic crisis, farmers across the country are plowing under their fields and laying off workers. Plant biomass has been shown by the DARPA “Blue Angel” project in 2010 to be an efficient way to rapidly make vaccines and diagnostics. This technology could pivot some areas of agriculture toward biomedical products to aid in the COVID-19 pandemic response.

## Introduction

**Figure 1:**
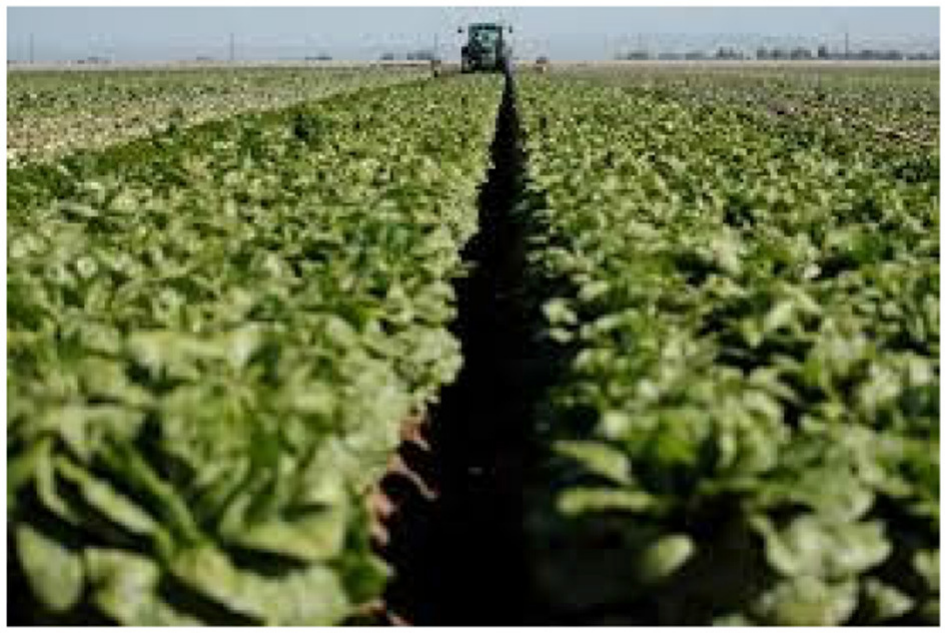
California farmer plows under lettuce after coronavirus shutters restaurant market, April 15, 2020. REUTERS/Mike Blake (Blake and Walljasper, 2020).

One of the alarming consequences of the COVID-19 pandemic crisis has been the devastating impact it has had on farms across the country, particularly at a time when unemployment has reached record highs, there are massive lines at food banks, and agricultural work is one of a limited number of jobs that is deemed essential. But many farms that provide produce to foodservice businesses such as restaurants, school cafeterias, hotels and cruise lines, have seen their orders vanish overnight as a result of shelter-in-place orders across the country and have plowed their fields under (Figure 1). Farmers can't afford to pay the labor, harvesting, and storage costs for a crop with no buyer.

At the same time, the world is facing a dramatic shortage of COVID-19 tests for both the SARS-CoV-2 virus responsible for the pandemic, as well as for antibodies that indicate whether a person has been infected by the virus in the recent past. COVID-19 tests are essential to determine who is currently infectious and who has recovered from the virus, yet two major roadblocks remain—an on-going shortage of the components needed to build these tests and a need to improve antibody test reliability. Getting past these roadblocks will allow us to reopen the country and the world. Not only do these tests need to be quickly developed, validated, and approved by the FDA, but they will need to be globally affordable and deployed at an unprecedented scale (on the order of tens of millions of tests per week, and expanding further as we move from humans to animals to environmental samples). How can new biotechnologies use harvested plant biomass to help address both crop surpluses and COVID-19 test shortages, while keeping farmers in business and farmworkers employed?

As a case study of the potential for plant-based biomanufacturing technologies to meet the described challenges, consider that on April 15th, 2020, a California farmer plowed under about 350 acres of romaine lettuce, valued at $1.46 million U.S dollars, and laid off 150–200 seasonal workers due to the loss of the restaurant and food service markets, which shut down as a result of COVID-19 (Blake and Walljasper, 2020). At a typical yield for romaine lettuce in Monterey County these 350 acres correspond to about 3 million kgs of fresh romaine biomass that was plowed under for this one farm. Rather than plowing this crop under, it could have been harvested and used to produce the proteins needed for making COVID-19 tests. DARPA made a $100 million investment in 2010 to demonstrate the large-scale production of therapeutic proteins in plants within contained biomanufacturing facilities to respond to pandemics. With the exception of the production of ZMapp antibodies to combat the 2013–2016 West Africa Ebola outbreak (Qiu et al., 2014) the technology has been fallow for this purpose. In the U.S., large scale plant-based recombinant protein production facilities have been built in Bryan, TX (Caliber Biotherapeutics, purchased by iBio CDMO) (Holtz et al., 2015), Owensboro, KY (Kentucky Bioprocessing, Inc.) (Pogue et al., 2010), and North Carolina (Medicago) (Lomonossoff and D'Aoust, 2016).

This technology could be used for rapid, flexible, scalable and cost-effective production of SARS-CoV-2 viral proteins, such as the full-length spike protein (in a stabilized, solubilized form), spike protein fragments (such as the receptor-binding domain, RBD), or the nucleocapsid protein. These proteins, called antigens, bind specifically to SARS-CoV-2 antibodies in serological tests in Enzyme Linked Immunosorbent Assay (ELISA) or Lateral Flow Assay (LFA) formats and can be used to test for the presence of these antibodies in human or animal samples. The soluble spike protein is a homotrimer comprised of 138 kDa highly glycosylated monomers, while the spike RBD is a 25 kDa fragment with only 2 glycosylation sites. For detection of the virus itself, proteins that bind to SARS-CoV-2 virus particles, such as the angiotensin converting enzyme-2 (ACE2) could also be produced in plants. Currently, these diagnostic reagents are being produced in mammalian cell culture (human embryonic kidney or HEK, Chinese Hamster Ovary or CHO, etc.), primarily using transient transfection processes due to the long times (several months to a year) required to develop stable mammalian cell lines with high titers. However, the yields are quite low [~5 mg/L for the full-length spike protein (Esposito et al., 2020) and >20 mg/L for the spike RBD (Stadlbauer et al., 2020)], and the processes require expensive media, transfection reagents, and expression enhancers, a source of mammalian cells with high viability, and have limited scalability. Depending on the host cell line and method used to introduce the DNA into the cells these processes may require more expensive facilities (Biosafety Level 2) capable of handling human infectious agents. We propose that using plant tissue to produce proteins for COVID-19 tests will be more cost-effective, faster, and scalable than traditional biomanufacturing approaches. As an added bonus, this technology benefits agricultural producers by providing a market for crops that would otherwise be destroyed.

## How Does the Plant-Made Antigen Production Technology Work?

In the transient agroinfiltration process, fresh (viable) plant biomass is submerged in a solution of genetically engineered bacteria, *Agrobacterium tumefaciens* (the vector used to introduce foreign DNA into the plant cells), containing an expression cassette for production of the target protein. In commercial scale facilities (Pogue et al., 2010; Holtz et al., 2015; Lomonossoff and D'Aoust, 2016), whole plants grown indoors in trays under artificial lighting (e.g., LEDs) are used as the production host, however transient agroinfiltration and recombinant protein production in harvested plant biomass has also been demonstrated (Joh et al., 2005; Plesha et al., 2009; Fujiuchi et al., 2016; Jung and McDonald, 2016). For whole plants, the trays are turned upside down and the aerial portions of the plants are submerged in the agrobacterial solution while harvested plant biomass can be directly submerged. A moderate vacuum is applied to remove air from the interstitial spaces of the leaves for a few minutes, then the vacuum is released allowing the agrobacterial solution to infiltrate throughout the leaf tissue. The biomass is then incubated under controlled conditions, typically 5–7 days, to allow time for the agrobacteria to transfer the DNA production instructions to the plant host cells which make the protein using the plant host cell's protein synthesis machinery. The plant biomass is then homogenized and the target protein is extracted, concentrated, and purified to the level required for the intended application. For diagnostic applications, an additional amino acid sequence (such as a 6-histidine tag) can be included at the end of the protein to simplify purification using an affinity chromatography column. Although the production yields using this technology are protein dependent, they can reach up to 1 g/kg fresh weight (FW) biomass (Lomonossoff and D'Aoust, 2016).

We have previously presented a technoeconomic model for large scale production of monoclonal antibody (mAb) therapeutic proteins produced in indoor-grown tobacco (*Nicotiana benthamiana*) plants (Nandi et al., 2016). This model is based on the Caliber Biotherapeutics data generated during full-scale production for the DARPA “Blue Angel” project in the facility described by Holtz et al. (2015). It can be used to provide a preliminary analysis of how a single facility like this could be used to produce SARS-CoV-2 diagnostic proteins in harvested lettuce. Table 1 shows results of the calculations assuming the romaine lettuce feedstock is processed at the same throughput as *N. benthamiana* plant tissue (9,830 kg biomass/batch, 1 batch/week) for varying production levels in lettuce (which are unknown at this time) ranging from 1 mg purified antigen/kg FW to 100 mg purified antigen/kg FW after purification, assuming the same protein loss (35%) in downstream processing as for the mAb case. For the modeled facility with 1 day for vacuum infiltration (assuming two vacuum infiltration chambers operating in parallel), a 7 day incubation, and 1 day purification, the overall batch time from infiltration through bulk formulation is about 9 days. Higher throughputs could be achieved by increasing the number of vacuum infiltration units and downstream purification trains and staggering batches to infiltrate biomass more frequently than once a week (e.g., daily). The cost of production of the antigen is assumed to be the same as the cost for processing one batch of *N. benthamiana* for mAb production [see Table 1 in Nandi et al. (2016)] and adding $1.5M in feedstock costs to pay for the lettuce, coming to $28.4M/year not including depreciation, or $600K/batch. Note that these production costs also do not include any costs for transporting the harvested biomass from the farm to the processing facility.

**Table 1:** Impact of antigen production yield on number of tests per batch, time to reach 1 billion tests, and cost of antigen per test for plant-made antigens.

**Production yield (mg purified antigen/kg FW)**	**1 mg/kg FW**	**50 mg/kg FW**	**100 mg/kg FW**
Antigen (g)/batch	9.83	492	983
Number of ELISA tests/batch @ 300 ng/test	32.8M	1,640M	3,280M
Number of LFA tests/batch @ 1 μg/test	9.83M	492M	983M
Time to reach 1 billion ELISA tests (months)	30.5	0.61	0.31
Time to reach 1 billion LFA tests (months)	100	2	1
Cost of antigen per ELISA test (cents/test)	2.00	0.04	0.02
Cost of antigen per LFA test (cents/test)	6.00	0.12	0.06

## Discussion

### The Advantage of Scale

Agriculture is inherently a scalable system, especially with field grown plants that only require light, water and fertilizer, but particularly in situations where markets vanish rapidly in a large-scale production infrastructure. Consider the fact that even with the lowest antigen production level (1 mg/kg FW) it would only require about 36 acres of lettuce to produce antigens for 1 billion ELISA tests and about 120 acres for 1 billion LFA tests. Given published yields for full spike proteins (Esposito et al., 2020) it would require 60,000 L of mammalian cell culture to produce antigen for 1 billion ELISA tests and 200,000 L of mammalian cell culture to produce antigen for 1 billion LFA tests. Although these scales are not unreasonably large, it should also be pointed out that there is also an urgent need for mammalian cell culture capacity for production of vaccines and therapeutics to treat COVID-19, in addition to the cost considerations described below. And while bioreactor working volumes up to 20,000 L are common for stable CHO suspension cultures, the largest scale that has been reported for transient transfection of mammalian cells is 100 L (Girard et al., 2002) so antigen production for 1 billion ELISA tests would require 600 mammalian bioreactors runs; 20,000 bioreactor runs would be needed to produce antigen for 1 billion LFA tests. As antigen production yields increase for mammalian cell transient transfection processes, the required culture volumes and number of bioreactor runs will decrease proportionately. Ultimately, stable CHO lines with high antigen titers will be available but it will take time to develop these lines and scale up production processes. To meet current societal needs for diagnostic and therapeutic proteins, all biomanufacturing platforms will be needed, including mammalian cell culture. Luckily, use of plant biomass has far better economies of scale and will be able to ramp up quickly to meet the 1 billion test target.

### The Advantage of Speed

Transient expression in plants is inherently fast, particularly when the plant biomass is already available, with an overall batch time, including purification, of about 9 days. To avoid transportation time and costs mobile vacuum infiltration units and prefabricated mobile cleanroom protein purification pods could be deployed onsite. Use of harvested plant biomass that is available saves a great deal of time compared with the time required to expand mammalian cells in culture for transfection and/or develop stable transgenic mammalian cell lines that express the antigen at high level. The overall batch time for transient transfection of mammalian cells at the 100 L bioreactor scale, not including downstream processing and purification, is about a month, including the time required to expand cells prior to transfection (Girard et al., 2002).

### The Advantage of Cost

Cost is one of the main advantages of the proposed approach with the antigen production contributing cents or fractions thereof to the cost of the test. One cost driver is that plants can be grown outdoors while mammalian cells must be grown under aseptic conditions (requiring either expensive single use disposable bioreactors or extensive cleaning and sterilization operations), as well as requiring expensive medium and transfection reagents. The cost estimates shown in Table 1 are an overestimate since they include costs associated with plant growth (not needed if harvested field grown biomass is used), as well as lighting during incubation (also not needed) and three chromatography steps which would not likely be needed for a diagnostic reagent. Waste biomass could be processed through anaerobic digestion to produce biogas energy to help power the facility as well as safe biofertilizer solids that could be returned to the farmer to offset raw materials costs. Assuming a mammalian cell culture media cost of $100/L, the cost of the medium alone, not including costs for labor, chromatography resins, buffers, utilities, etc., would contribute 0.6 cents per ELISA test and 2 cents per LFA at the stated expression level for the full spike protein of 5 mg/L. Our previous work has shown that annual production costs for monoclonal antibody production using the transient plant system are about half of the production costs for stable CHO mAb production facilities at similar production scales (Nandi et al., 2016). We expect the cost advantage to be even more significant compared with mammalian cell culture transient transfection processes and use of lower cost field-grown plant biomass.

### The Advantage of Flexibility

There is growing evidence that the SARS-CoV-2 spike protein is mutating so there may be a need for redesigned antigens for detection of both the virus and circulating antibodies. With the transient plant-based system, it is easy to change to produce a new antigen by introducing newly engineered agrobacteria that express the optimized antigen. Antigens are also decorated with sugars in a process known as glycosylation and these sugars can impact their binding and detection of antibodies that indicated prior exposure to SARS-CoV-2. Fortunately, plant glycosylation can be altered as needed through both process engineering approaches (Castilho et al., 2010; Kommineni et al., 2019) and/or post processing modifications (Bennett et al., 2018), although the latter would add some additional cost.

### Challenges, Innovations Needed, and Opportunities

Large multimeric glycoproteins are challenging to produce in any system and it is not clear, particularly for the full soluble spike trimer, whether expression levels of 100 mg/kg or greater can be obtained without significant process optimization. As shown in Table 1, production yield plays a critical role in both antigen manufacturing costs and time to produce enough antigen for 1 billion tests. Another potential concern is feedstock variability associated with the use of field-grown plants which could introduce process challenges to ensure product quality and consistency. But field-grown plants have been used for medical applications for many years, for example the antimalarial drug artemisinin is still primarily obtained from field-grown *Artemisia annua* (Ikram and Simonsen, 2017). In addition, field-grown plants have also been used for transient production of therapeutics and vaccines. Over 25 years ago, the company Large Scale Biology, Inc. pioneered the use engineered plant viral vectors for transient production of therapeutic proteins and vaccines in field-grown tobacco, with extraction and purification of the biologics from the harvested plant tissue taking place in their indoor facility (Pogue et al., 2002). In the scenario we propose for SARS-CoV-2 antigen production, transient agroinfiltration of harvested plant biomass would take place in an indoor facility, along with the extraction and purification of the antigen. While transient expression of recombinant proteins using agroinfiltration of harvested biomass has been demonstrated by our group and others at the lab scale in a variety of plants including *N. benthamiana* (Plesha et al., 2009; Fujiuchi et al., 2016), sunflower (Jung and McDonald, 2016), and lettuce (Joh et al., 2005), it has not yet been established at large scale. Additional work is needed to establish post-harvesting and post-infiltration handling and process conditions, as well as efficient expression systems for lettuce and other green leafy crops that are amenable to vacuum agroinfiltration. Equipment modifications and redesign will also be needed. For example, vacuum infiltrators will need to be redesigned/adapted to handle harvested biomass and process equipment and environmental controls for humidity and temperature during incubation will need to be established to preserve leaf health and protein production during the incubation phase. But, just as during World War II when large-scale stirred, aerated fungal fermenters were developed for the production of antibiotics by collaborative efforts of microbiologists and engineers (ultimately leading to the industrial fermentation industry), this could be the time to bring together agriculture and biomanufacturing. It requires us to think creatively when it comes to how we make biologics, whether it is for industrial enzymes, biomaterials, diagnostics or therapeutics, perhaps broadening the plant-made proteins (PMP) industry. This could also improve rural America's trust in science (Krause et al., 2019), help keep farms in business and farmworkers employed during the COVID-19 pandemic, and prepare us to respond to future outbreaks.

## Author Contributions

KM conceived the topic and drafted the article. RH provided industrial input and reviewed and edited the article. Both authors contributed to the facility techno-economic model that the analysis is based on.

## Conflict of Interest

RH is currently the Principal of Holtz Biopharma Consulting and was previously employed by iBio CDMO, LLC (formerly Caliber Biotherapeutics). KM is a cofounder of Inserogen, Inc. Any opinions, findings, and conclusions or recommendations expressed in this article are those of the authors and do not necessarily reflect the views of Holtz Biopharma Consulting, iBio CDMO, LLC or Inserogen, Inc.
